# Supplementary figures and images for: Gut microbiota promotes host resistance to low-temperature stress by stimulating its arginine and proline metabolism pathway in adult Bactrocera dorsalis
Source: PLoS Pathog. 2020 Apr 15;16(4):e1008441. doi: 10.1371/journal.ppat.1008441 (PMC7185725; doi:10.1371/journal.ppat.1008441)

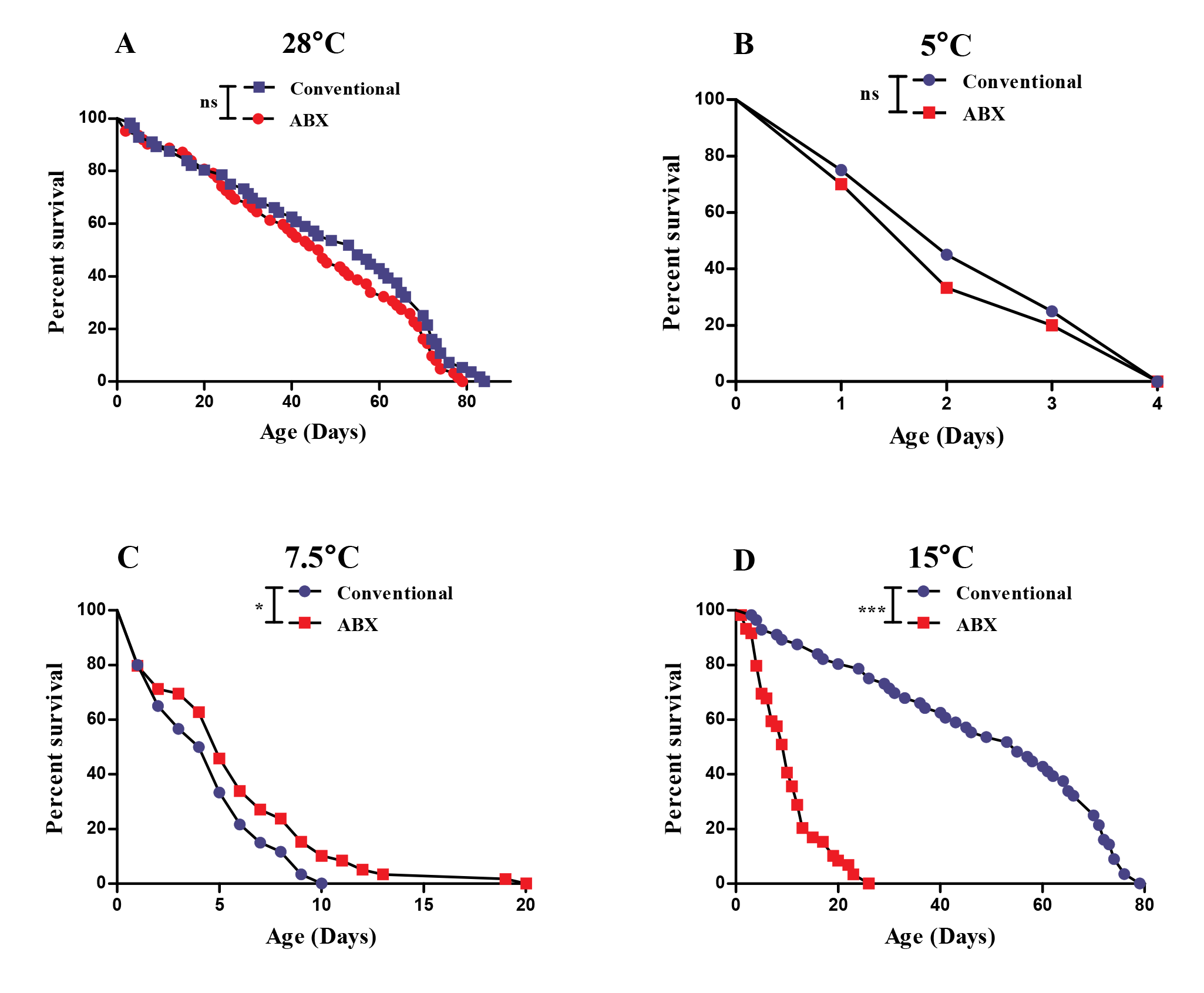

Supplement: S1 Fig — (A) There was a nonsignificant difference in the survival time of ABX and conventional flies under normal rearing temperature (28°C). (B) A nonsignificant difference was observed between median survival time of ABX (2 days) and conventional (2 days) flies at 5°C. (C) Under 7.5°C temperature stress, there was a small but statistically significant percent survival change, but median survival time was not significantly different between ABX (5 days) and conventional (4.5 days) flies. (D) Conventional fly median survival time was enhanced at 15°C (55 days) compared with that of ABX flies (10 days). Newly eclosed flies were treated with antibiotic solution for 5 consecutive days to generate ABX flies, while conventional flies were only fed sterile liquid diet. After 5 days posteclosion, both group of flies were fed with sterile liquid diet until mortality. n = 60 for each condition, log-rank (Mantel-Cox) test, *** P<0.0001, *P<0.05 and ns P>0.05). (TIF) [file ppat.1008441.s001.tif]

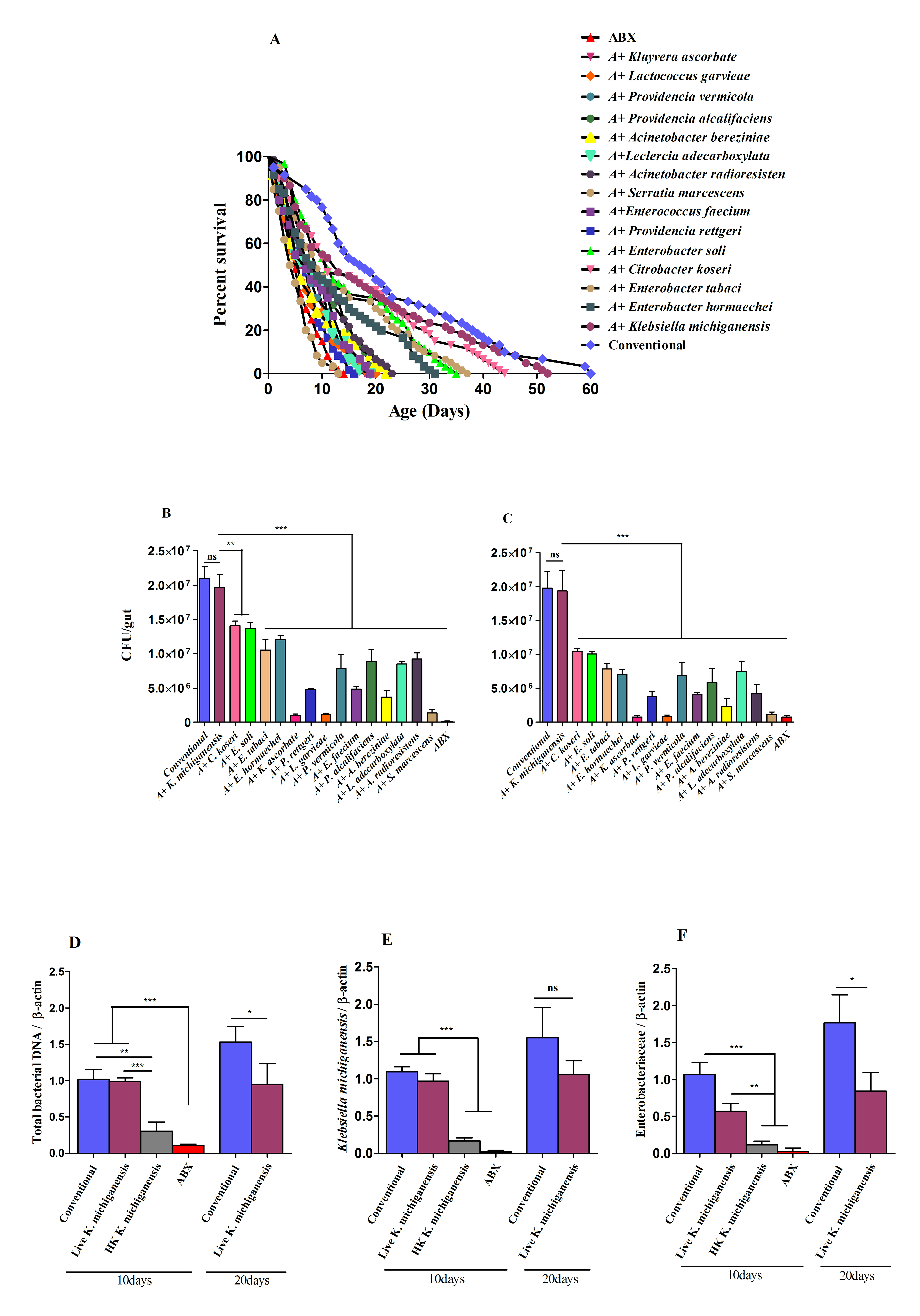

Supplement: S2 Fig — (A) The median survival time was extended in ABX flies recolonized with the following gut symbionts (P<0.05): K. michiganensis (13 days), E. soli and C. koseri (11 days), E. tabaci (9 days), E. hormaechei (8.5 days), A. radioresistens and P. alcalifaciens (8 days), Leclercia adecarboxylata (7.5 days), Enterococcus faecium and P. vermicola (7 days), P. rettgeri (6.5 days) and A. bereziniae (6 days) compared with 5 days in the ABX control. In contrast, median survival time was unaffected (P>0.05) after the recolonization of ABX flies with Lactococcus garvieae, Serratia marcescens and Kluyvera ascorbata compared with 5 days in the ABX control. Note: There were significant differences (P<0.05) in the median survival time between conventional flies and all other tested strains, except in conventional vs. K. michiganensis-reinfected flies (P = 0.1275), under low-temperature stress of 10°C. Log-rank (Mantel-Cox) test was used to compare the survival between different treatments. *A+ strain name (ABX flies recolonized by the above gut bacterial strains). (B and C) Microbial load in gut homogenates is maintained at different ages after feeding live K. michiganensis to ABX flies and in conventional flies. (B) Colony-forming units (CFUs) of flies after 5 dpe to 10°C; the average number of cultivated microbial communities resulting from CFU in ABX control fed with sterile liquid diet was 1 x10^5^ ± 5.7 x 10^4^ CFUs gut-1 (mean ± SE of 10 individual flies), representing 99.49% decreases vs. that of live K. michiganensis-reinfected flies, which showed 1.97 x 10^7^ ± 1.87 x 10^6^ CFUs gut-1 (mean ± SE of 10 individual flies) (P<0.0001). No significant difference was observed in the average number of cultivated microbial communities between conventional (2.103 x 10^7^ ± 1.637 x 10^6^ CFUs gut-1) and live K. michiganensis-reinfected (1.97 x 10^7^ ± 1.87 x 10^6^ CFUs gut-1) (P>0.05) flies at 5 dpe to 10°C. The average numbers of cultivated microbial communities resulting from CFUs after 5 dpe to 10° [file ppat.1008441.s002.tif]

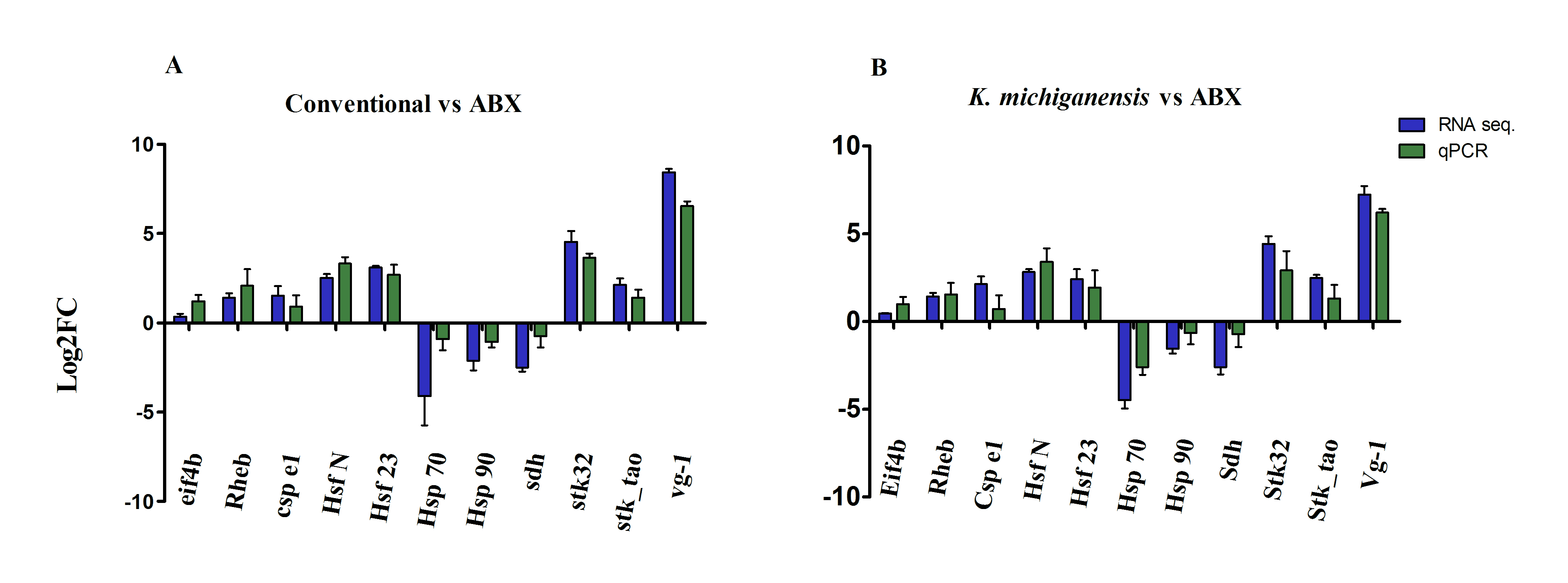

Supplement: S3 Fig — Data are presented in Log2Fold Change. In the presence of gut microbes, some of these HSP, ZFP and STK transcripts were also downregulated in those comparison groups, while the transcripts related to arginine and proline metabolism were only upregulated in those group comparisons in response to low-temperature stress and overlapped in both omics approaches. Therefore, arginine and proline pathway was chosen to explore the low-temperature stress resistance. * Target of rapamycin genes (Eif4b, Rheb), protein processing in endoplasmic reticulum (cold shock protein e-1: csp e1; heat shock protein family: Hsf N, Hsf 23, Hsp90, Hsp70; serine/threonine kinase proteins: Stk32, Stk Tao; saccharopine dehydrogenase: Sdh; Vitellogenin-1: Vg-1). (TIF) [file ppat.1008441.s003.tif]

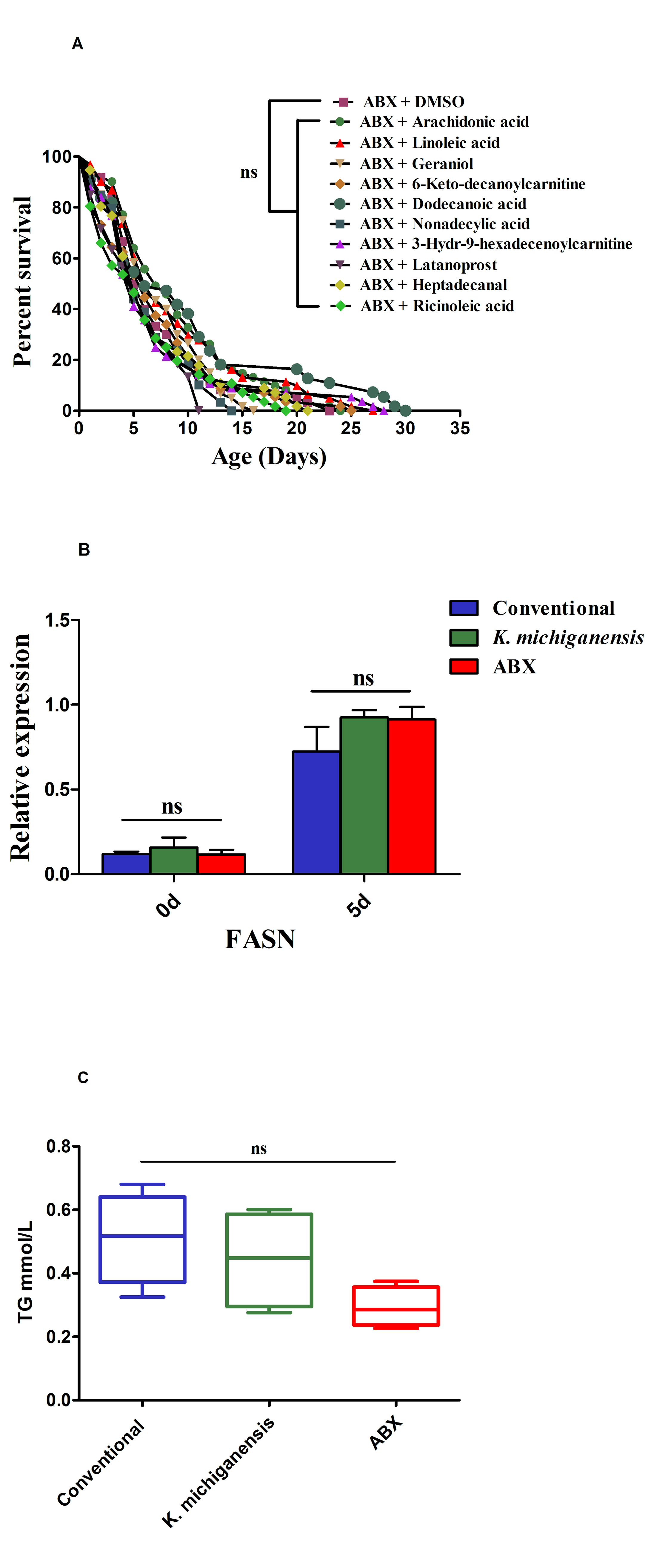

Supplement: S4 Fig — (A) Survival curves at low-temperature stress of 10°C, microinjection of arachidonic acid (P = 0.1005), linoleic acid (P = 0.0916), Geraniol (P = 0.9355), 6-Keto-decanoylcarnitine (P = 0.9643), Dodecanoic acid (P = 0.5675), Nonadecylic acid (P = 0.2949), 3-Hydroxy-9-hexadecenoylcarnitine (P = 0.9518), Latanoprost (P = 0.0547), Heptadecanal (P = 0.5722) and Ricinoleic acid (P = 0.1754) to ABX flies compared with ABX control injected with DMSO (n = 60 for each condition, log-rank test, P>0.05 denoted as nonsignificant (ns). (B) FASN gene expression was not stimulated during low-temperature stress. The relative expression levels of fatty acid synthase (FASN) gene, a key enzyme that catalyzes the reductive synthesis of long-chain fatty acids (e.g., arachidonic acid, linoleic acid) at 0 day and 5 dpe to 10°C in conventional, K. michiganensis-reinfected and ABX flies. One-way analysis of variance (ANOVA) and Tukey’s test were performed. Statistical significance was indicated as follows: P>0.05 denoted as nonsignificant (ns). (C) TG levels were not significantly different in the fly fat body tissues among conventional, K. michiganensis-reinfected or ABX treatments (One-way analysis of variance (ANOVA) and Tukey’s test were performed. Statistical significance was indicated as follows: P>0.05 denoted as nonsignificant (ns). (TIF) [file ppat.1008441.s004.tif]

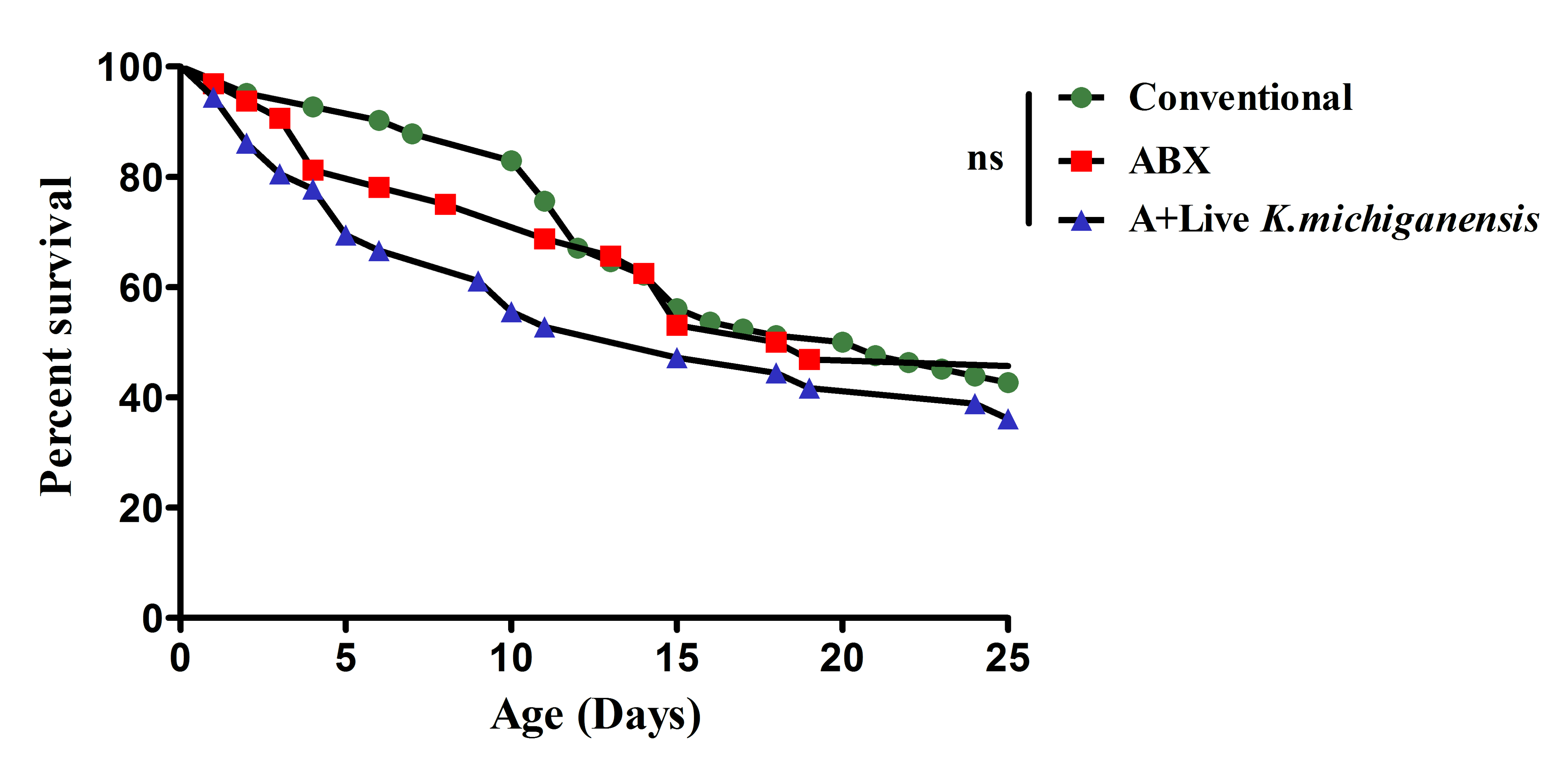

Supplement: S5 Fig — The median survival time for conventional and ABX flies fed with K. michiganensis were 20.50 and 15 days, respectively, compared with 18.50 days found in the ABX flies. These results showed that there were non-significant differences in the median survival time among different treatments, suggesting that gut bacteria e.g., K. michiganensis might not have symbiotic effect to provide resistance against UV stress (n = 60 for each condition, log-rank test, P>0.05 denoted as nonsignificant (ns). (TIF) [file ppat.1008441.s005.tif]
