# Supplementary material for: Gut microbiota promotes host resistance to low-temperature stress by stimulating its arginine and proline metabolism pathway in adult Bactrocera dorsalis
Source: PLoS Pathog. 2020 Apr 15;16(4):e1008441. doi: 10.1371/journal.ppat.1008441 (PMC7185725; doi:10.1371/journal.ppat.1008441)
Supplement: S1 Text — (DOCX) [file ppat.1008441.s009.docx]

**S1 Methods**

**RNA sequencing**

RNA sequencing was performed in three libraries of ABX (ABX-1, ABX-2 and ABX-3), conventional (Conv-1, Conv -2, and Conv-3) and *K. michiganensis*-reinfected flies (*K. michiganensis****-***1, *K. michiganensis****-***2 and *K. michiganensis****-***3).

**RNA quantification and qualification**

RNA degradation and contamination was monitored on 1% agarose gels. The purity was checked using the NanoPhotometer® spectrophotometer (IMPLEN, CA, USA). The concentration was measured using Qubit® RNA Assay Kit in Qubit® 2.0 Flurometer (Life Technologies, CA, USA).

RNA integrity was assessed using the RNA Nano 6000 Assay Kit of the Agilent Bioanalyzer 2100

system (Agilent Technologies, CA, USA).

**Library preparation for Transcriptome sequencing**

From each sample, 1.5 μg RNA was employed as input material for the preparation of RNA samples. Sequencing libraries were created using a NEBNext® Ultra™ RNA Library Prep Kit for Illumina® (NEB, USA) according to the manufacturer’s instructions. The purification of mRNA was performed from total RNA using poly-T oligo-attached magnetic beads. First-strand cDNA was synthesized by using hexamer primer and M-MuLV Reverse Transcriptase (RNase H-). The library fragments were purified with an AMPure XP system (Beckman Coulter, Beverly, USA) to allow the selection of 250~300 bp cDNA fragments. Prior to PCR, 3 μl USER Enzyme (NEB, USA) was used with size-selected and adapter-ligated cDNA at 37°C for 15 minutes, followed by 5 minutes at 95°C. Subsequently, PCR was performed with Phusion high-fidelity polymerase and universal PCR primers. Finally, PCR products were purified (AMPure XP system) followed by library quality assessment on the Agilent Bioanalyzer 2100 system.

**Clustering and sequencing (Novogene Experimental Department)**

The clustering of the index-coded samples was performed on a cBot Cluster Generation System using TruSeq PE Cluster Kit v3-cBot-HS (Illumia) according to the manufacturer’s instructions. After cluster generation, the library preparations were sequenced on an Illumina Hiseq platform and paired-end reads were generated.

**Quality control**

Raw data (raw reads) of fastq format were firstly processed through in-house perl scripts. In this step, clean data(clean reads) were obtained by removing reads containing adapter, reads containing ploy-N and low quality reads from raw data. At the same time, Q20, Q30, GC-content and sequence duplication level of the clean data were calculated (Table 1). All the downstream analyses were based on clean data with high quality.

**Gene functional annotation**

Gene function was annotated based on the following databases: Nr (NCBI non-redundant protein sequences); Nt (NCBI non-redundant nucleotide sequences); Pfam (Protein family) KOG/COG (Clusters of Orthologous Groups of proteins); Swiss-Prot (A manually annotated and reviewed protein sequence database); KO (KEGG Ortholog database); GO (Gene Ontology) [1].

**Differential expression analysis**

Differential expression analysis of two group comparisons (conventional vs. ABX and *K. michiganensis****-***reinfected vs. ABX) was performed using the DESeq R package (1.10.1). DESeq provide statistical routines for determining differential expression in digital gene expression data using a model based on the negative binomial distribution [2]. The resulting P values were adjusted using the Benjamini and Hochberg’s approach for controlling the false discovery rate. Genes with an adjusted P-value <0.05 found by DESeq were assigned as differentially expressed [3].

**GO enrichment analysis**

Gene Ontology (GO) enrichment analysis of the differentially expressed genes (DEGs) was implemented by the GOseq R packages based Wallenius non-central hyper-geometric distribution [4], which can adjust for gene length bias in DEGs.

**KEGG pathway enrichment analysis**

We used KOBAS software to test the statistical enrichment of differential expression genes in KEGG pathways. [5, 6]

| Table 1. Statistics for filtering and mapping reads | | | | | | | | | |
| --- | --- | --- | --- | --- | --- | --- | --- | --- | --- |
| **Sample** | **Raw Reads** | **Clean Reads** | **Clean reads (%)** | **Clean bases** | **Error (%)** | **Q20(%)** | **Q30(%)** | **GC (%)** |  |
| ABX-1 | 46130628 | 45649708 | 98.96 | 6.85G | 0.02 | 98.14 | 94.41 | 42.5 |  |
| ABX-2 | 45532684 | 44642546 | 98.05 | 6.7G | 0.02 | 98.23 | 94.6 | 41.98 |  |
| ABX-3 | 57199854 | 56663652 | 99.06 | 8.5G | 0.03 | 97.87 | 93.77 | 41.94 |  |
| Conventional 1 | 53490556 | 53047534 | 99.17 | 7.96G | 0.02 | 98.12 | 94.34 | 41.58 |  |
| Conventional 2 | 51484620 | 51088442 | 99.23 | 7.66G | 0.03 | 97.94 | 93.89 | 41.44 |  |
| Conventional 3 | 57768904 | 57305644 | 99.2 | 8.6G | 0.03 | 97.94 | 93.91 | 41.68 |  |
| *K. michiganensis* 1 | 50532376 | 50217076 | 99.38 | 7.53G | 0.02 | 98.15 | 94.41 | 42.17 |  |
| *K. michiganensis* 2 | 48637002 | 48325896 | 99.36 | 7.25G | 0.02 | 98.16 | 94.43 | 41.68 |  |
| *K. michiganensis* 3 | 55559126 | 55224352 | 99.4 | 8.28G | 0.03 | 97.94 | 93.89 | 42.03 |  |

**Real-time PCR.** For gene expression analysis, 10 flies (sex ratio 1:1) were collected for RNA extraction at different time intervals. RNAiso Plus reagent (Takara, Japan) was used for RNA extraction. cDNA synthesis was performed using 500 ng total RNA with Transcript RT Master Mix (Takara, Japan) according to the manufacturer’s guidelines. RT-qPCR was performed on a BioRad MyIQ2 instrument using BioRad SYBR Green qPCR mix (BioRad, USA). All samples were analyzed in triplicate (technical repeats). The reactions included 5 μl SYBR Green mix, 3.20 μl ddH_2_O, 1 μl cDNA and 0.4 μl each of forward and reverse primers. The amplification program included preincubation at 95°C for 30 seconds, followed by 45 cycles of denaturation at 95°C for 15 seconds and annealing at 60°C for 30 seconds. At the end of each amplification run, melting curve analysis was performed to confirm the presence of a single peak. For the melting curve analysis, the thermocycler conditions were 55°C for 60 seconds, followed by 81 cycles beginning at 55°C for 10 seconds, with a 0.5°C increase each cycle [58]. The levels of the detected mRNA by cycling threshold analysis were normalized using alpha-tubulin or rpl32 (S1 Table) as the control.

Table 2. Quantification of distorted mitochondria percentage

| **Treatment groups** | **Conventional** | **ABX** | ***K. michiganensis*** |
| --- | --- | --- | --- |
| Number of flies | 6 | 6 | 6 |
| Total number of gut cell images counted | 60 | 56 | 63 |
| Total number of mitochondria counted in all cell images | 1071 | 1132 | 1316 |
|  |  |  |  |

*This data is presented in figure 9 (D) after statistical analyses.

**References**

1. Anders S, Huber W. Differential expression analysis for sequence count data. Gen Biol. 2010. doi:10.1186/gb-2010-11-10-r106.
2. Finn RD, Tate J, Mistry J, et al. The Pfam protein families database. Nuc A Res. 2008; 36, D281–D288.
3. Mao X, Cai T, Olyarchuk JG, et al. Automated genome annotation and pathway identification using the KEGG Orthology (KO) as a controlled vocabulary. Bioinformatics. 2005; 21: 3787–3793.
4. Kanehisa M, Araki M, Goto S, et al. KEGG for linking genomes to life and the environment. Nuc A res. 2008; 36: D480-D484.
5. Wang L, Feng Z, Wang X, et al. DEGseq: an R package for identifying differentially expressed genes from RNA-seq data. Bioinf. 2010; 26: 136-138.
6. Li B, Dewey C. RSEM: accurate transcript quantification from RNA-Seq data with or without a reference genome. BMC Bioinf. 2011. doi:10.1186/1471-2105-12-323.
