# Supplementary material for: Gut microbiota promotes host resistance to low-temperature stress by stimulating its arginine and proline metabolism pathway in adult Bactrocera dorsalis
Source: PLoS Pathog. 2020 Apr 15;16(4):e1008441. doi: 10.1371/journal.ppat.1008441 (PMC7185725; doi:10.1371/journal.ppat.1008441)
Supplement: S1 Table — (A) Primers used for PCR amplification to target the bacterial 16S rRNA gene, quantification (qPCR). * indicates the reference numbers. (B) The relative abundances of 15 representative bacterial strains isolated from the guts conventional flies 5 dpe to 28°C and 10°C. *The strains isolated from the gut of B. dorsalis in this study are designated with numbers, e.g., BD473 according to our previous publication, with only one new strain Serratia marcescens isolated in this study represented as BD164. (DOCX) [file ppat.1008441.s010.docx]

S1 Table A. Primers used for PCR amplification to target the bacterial 16S rRNA gene, quantification (qPCR).

| **Primer** | **Primer sequence (5′–3′)** | **Fwd/Rev** | **Use** | **Reference** |
| --- | --- | --- | --- | --- |
| ODC | TACGAAAAAGCGACCAGGCT | Fwd | real time PCR | This study |
| ODC | CCGAGTCACCTCCAGTATGC | Rev | real time PCR | This study |
| Arg | CGGTGTGGACCCAATACCAA | Fwd | real time PCR | This study |
| Arg | AAAGATTGGGCGTGTGTTGC | Rev | real time PCR | This study |
| ASS | TGTCCTCGAAATCGAACGGT | Fwd | real time PCR | This study |
| ASS | TCAGCTGACAGGTCTGGCTA | Rev | real time PCR | This study |
| Oat | AGAAAATAAATACGGCGCACACA | Fwd | real time PCR | This study |
| Oat | GCTTCTTGGCTTGCTCTGTC | Rev | real time PCR | This study |
| proC | GCTAGACGGTAATTGAGCCCT | Fwd | real time PCR | This study |
| proC | TACTGGCCGAGCTGTTAAGC | Rev | real time PCR | This study |
| *Klebsiella michiganensis* | CAGCCACACTGGAACTGAGA | Fwd | real time PCR | Sudakaran et al. 2012^*1^ |
| *Klebsiella michiganensis* | GTTAGCCGGTGCTTCTTCTG | Rev | real time PCR | Sudakaran et al. 2012^*1^ |
| uni331 | TCCTACGGGAGGCAGCAGT | Fwd | real time PCR | Nadkarni et al. 2002^*2^ |
| uni797 | GGACTACCAGGGTATCTATCCTGTT | Rev | real time PCR | Nadkarni et al. 2002^*2^ |
| β -actin | TCGATCATGAAGTGCGATGT | Fwd | real time PCR | Yao et al. 2016^*60^ |
| β -actin | ATCAGCAATACCGGGGTACA | Rev | real time PCR | Yao et al. 2016^*60^ |
| En-lsu-3 | TGCCGTAACTTCGGGAGAAGGCA | Fwd | real time PCR | Matsuda et al. 2009^*3^ |
| En-lsu-3 | TCAAGGACCAGTGTTCAGTGTC | Rev | real time PCR | Matsuda et al. 2009^*3^ |
| α-tubulin | CGCATTCATGGTTGATAACG | Fwd | real time PCR | Suganya et al. 2010^*4^ |
| α-tubulin | GGGCACCAAGTTAGTCTGGA | Rev | real time PCR | Suganya et al. 2010^*4^ |
| Hsp70 | ATCTACTTCCTCAACGGTGGGT | Fwd | real time PCR | This study |
| Hsp70 | GACAAAACTACATAGTGGTGGCG | Rev | real time PCR | This study |
| HSP23a | TGTTGGTTGGGTAAGGTCGAA | Fwd | real time PCR | This study |
| HSP23a | CCCAAACATCCCCTAACGCA | Rev | real time PCR | This study |
| HSFN a | CGTTTTAAACCAGCCTGGACG | Fwd | real time PCR | This study |
| HSFN a | CGCATGTCCTTCGACGATGT | Rev | real time PCR | This study |
| SDH | AGCGAGAGATCTTTGCCAACT | Fwd | real time PCR | This study |
| SDH | ACGGTACCGCAGTAATTTCA | Rev | real time PCR | This study |
| CSP e1 | GGTTCCGACTTCTCGCGATT | Fwd | real time PCR | This study |
| CSP e1 | GTAGAAGGGCCATTTGTTGGC | Rev | real time PCR | This study |
| STK32 | GACGGCGACGAATGGAAATG | Fwd | real time PCR | This study |
| STK32 | GCTCCACCCAGAATGGTTGT | Rev | real time PCR | This study |
| TAO | ACTGAAGCAGAGGAAAGGAACA | Fwd | real time PCR | This study |
| TAO | CATTTGACTGGCGAACGAGC | Rev | real time PCR | This study |
| Vg-1 | CAACCAATCAGCAATAACCAGGAC | Fwd | real time PCR | Yue et al. 2018^*5^ |
| Vg-1 | GCATCACCACGAGCCAAACC | Rev | real time PCR | Yue et al. 2018^*5^ |
| EIF4B | ATGGGATGCATAGAGACGTTTA | Fwd | real time PCR | This study |
| EIF4B | ACTGAAAATCCGAACGCAGG | Rev | real time PCR | This study |
| RHEB | TGTTAGGATTTCCTGACTTTCCT | Fwd | real time PCR | This study |
| RHEB | TGAAGAAGGCGTTTCTTTCACA | Rev | real time PCR | This study |
| FASN | ACAGAATAGCGTATGTTACCAGT | Fwd | real time PCR | Dong et al. 2016^*62^ |
| FASN | GCAACAGGTCCCACGTTTTC | Rev | real time PCR | Dong et al. 2016^*62^ |
| rpl32 | CCCGTCATATGCTGCCAACT | Fwd | real time PCR | Yao et al. 2016^*60^ |
| rpl32 | GCGCGCTCAACAATTTCCTT | Rev | real time PCR | Yao et al. 2016^*60^ |
| 27F | GTTTGATCCTGGCTCAG | Fwd | PCR | Ceja-Navarro et al. 2015^*6^ |
| 1492R | GGTTACCTTGTTACGACTT | Rev | PCR | Ceja-Navarro et al. 2015^*6^ |
| dsASS | GGATCCTAATACGACTCACTATAGGCGTCGCACCCGAATCTTT | Fwd | RNAi | This study |
| dsASS | GGATCCTAATACGACTCACTATAGGTCCCGATCTAAGCAGAACACTT | Rev | RNAi | This study |
| dsArg | GGATCCTAATACGACTCACTATAGGGGTTAGTGCCGCTACTCGAA | Fwd | RNAi | This study |
| dsArg | GGATCCTAATACGACTCACTATAGGTAGCGAAAGATTGGGCGTGT | Rev | RNAi | This study |
| dsproC | GGATCCTAATACGACTCACTATAGGGCACATGTGAAGAAGTGCCG | Fwd | RNAi | This study |
| dsproC | GGATCCTAATACGACTCACTATAGGGGCAACTGTTCCACGAAACC | Rev | RNAi | This study |
| dsODC | GGATCCTAATACGACTCACTATAGGTGCCAAATCCGACGACATCA | Fwd | RNAi | This study |
| dsODC | GGATCCTAATACGACTCACTATAGGCGGAGCCAACGTGAAAACTG | Rev | RNAi | This study |
| dsEGFP | GGATCCTAATACGACTCACTATAGGACGTAAACGGCCACAAGTTC | Fwd | RNAi | This study |
| dsEGFP | GGATCCTAATACGACTCACTATAGGAAGTCGTGCTGCTTCATGTG | Rev | RNAi | This study |

* indicates the reference numbers

**Supporting References**

1. Sudakaran S, Salem H, Kost C, Kaltenpoth M. Geographical and ecological stability of the symbiotic mid‐gut microbiota in European firebugs, *Pyrrhocoris apterus* (Hemiptera, Pyrrhocoridae). Mol Ecol. 2012; 21:6134-6151. <https://doi.org/10.1111/mec.12027>.

2. Nadkarni MA, Martin FE, Jacques NA, Hunter N. Determination of bacterial load by real-time PCR using a broad-range (universal) probe and primers set. Microbiology. 2002; 148:257-266. [https://doi:10.1099/00221287-148-1-257.](https://dx.doi.org/10.1099/00221287-148-1-257)

3. Matsuda K, Tsuji H, Asahara T, Kado Y, Nomoto K. Sensitive quantitative detection of commensal bacteria by rRNA-targeted reverse transcription-PCR. Appl Environ Microbiol. 2007; 73:32-39. <https://doi/10.1128/AEM.01224-06.>

4. Suganya R, Chen SL, Lu KH. Target of rapamycin in the oriental fruit fly *Bactrocera dorsalis* (Hendel): its cloning and effect on yolk protein expression. Arc insect biochem physiol. 2010; 75:45-56. https://doi.org/10.1002/arch.20383.

5. Yue Y, Yang R-L, Wang W-P, Zhou Q-H, Chen E-H, Yuan G-R, Wang J-J, Dou W. Involvement of Met and Kr-h1 in JH-mediated reproduction of female *Bactrocera dorsalis* (Hendel). Front Physiol. 2018; 9. <https://doi.org/10.3389/fphys.2018.00482>.

6. Ceja-Navarro JA, Vega FE, Karaoz U, Hao Z, Jenkins S, Lim HC, Kosina P, Infante F, Northen TR, Brodie EL. Gut microbiota mediate caffeine detoxification in the primary insect pest of coffee. Nat com. 2015; 6:7618. <http://doi.org/10.1038/ncomms8618>.

S1 Table B. The relative abundances of 15 representative bacterial strains isolated from the guts conventional flies 5 dpe to 28°C and 10°C.

| **Sr.#** | **Representative bacterial strain isolated from Conventional flies** | **5 dpe to 28°C** | **5 dpe to 10°C** | **Percentage change (10°C -28°C) /28°C** |
| --- | --- | --- | --- | --- |
| 1 | *Enterobacter soli* BD473* | 57 | 51 | -10.53 |
| 2 | *Enterobacter tabaci* BD138 | 37 | 42 | 13.51 |
| 3 | *Klebsiella michiganensis* BD177 | 33 | 29 | -12.12 |
| 4 | *Enterobacter hormaechei* BD188 | 33 | 30 | -9.09 |
| 5 | *Citrobacter koseri* BD195 | 35 | 25 | -28.57 |
| 6 | *Kluyvera ascorbate* BD180 | 9 | 12 | 33.33 |
| 7 | *Providencia rettgeri* BD171 | 7 | 11 | 57.14 |
| 8 | *Lactococcus garvieae* BD480 | 1 | 2 | 100 |
| 9 | *Providencia vermicola* BD232 | 1 | 2 | 100 |
| 10 | *Enterococcus faecium* BD402 | 1 | 1 | 0 |
| 11 | *Providencia alcalifaciens* BD401 | 1 | 2 | 100 |
| 12 | *Acinetobacter bereziniae* BD101 | 2 | 3 | 50 |
| 13 | *Leclercia adecarboxylata* BD183 | 1 | 4 | 300 |
| 14 | *Acinetobacter radioresistens* BD638 | 2 | 2 | 0 |
| 15 | *Serratia marcescens* BD164 | 3 | 1 | -66.67 |
|  |  | 223 | 217 |  |

*The strains isolated from the gut of *B. dorsalis* in this study are designated with numbers, e.g., BD473 according to our previous publication [32], with only one new strain *Serratia marcescens* isolated in this study represented as BD164.
