# Supplementary material for: Gut microbiota promotes host resistance to low-temperature stress by stimulating its arginine and proline metabolism pathway in adult Bactrocera dorsalis
Source: PLoS Pathog. 2020 Apr 15;16(4):e1008441. doi: 10.1371/journal.ppat.1008441 (PMC7185725; doi:10.1371/journal.ppat.1008441)
Supplement: Attachment — Submitted filename: Response letter ppathogens.doc [file ppat.1008441.s011.doc]

**Institute of Urban and Horticultural Entomology**

**College of Plant Science and Technology**

**Wuhan 430070, Hubei**

**P. R. China**

**Tel: 0086-27-8728-6962**

**Fax: 0086-27-8728-0796**

**华 中 农 业 大 学**

**Huazhong Agricultural University**

Janurary 19, 2020

To The Editor

PLoS Pathogens

Dear Sir/Madam,

Thank you for your mail concerning our manuscript entitled “**Gut Microbiota Promotes Host Resistance to Low-Temperature Stress by Stimulating its Arginine and Proline Metabolism Pathway in Adult *Bactrocera dorsalis***”, for consideration to **PLoS Pathogens.**

The remarks of both reviewers and editors were taken into account and were discussed extensively one by one with the co-authors. We have proofed and revised the manuscript carefully. All the major comments such as, a more in depth experiments related to beneficial aspects of dead microbes that are continually given throughout the life and their effects on survival, observation of the gut tissue mitochondrial morphology through transmission electron microscopy (TEM) and ATP levels among the different treatments, verification of the combined effect of key genes and metabolites under low- temperature stress, incorporation of suggested literature etc., have been considered carefully and corrected according to the reviewer’s suggestion. All minor comments were also modified and incorporated into the revision. Additionally, the data of *K. michiganensis* BD177 genome is referenced in publicly accessible database 'NCBI' with Accession ID: PRJNA602959.

In accordance with both reviewers and editor’s remarks, the manuscript has now been extensively revised to conform to guidelines for publication in **PLoS Pathogens**. It has also been revised again by the team at American journal experts (AJE) for correct English usage.

Our answers (in red) to **each comment** of the reviewers can be found in the following pages and revised draft.

We do hope that you will now find our revised manuscript suitable for publication in PLoS Pathogens journal in its present form.

Thanking you in anticipation of favorably considering our manuscript.

Yours sincerely,

Hongyu Zhang Ph.D. Professor (*On the behalf of all authors*)

**Additional comments by Editors**

We noticed that you used “data not shown”/"unpublished data" in the manuscript, we do not allow these references. The PLOS data access policy requires that all data be either published with the manuscript or made available in a publicly accessible database. Please amend the supplementary material to include the referenced data or remove the references. Please make a note of this change in your response to reviewers.

Thank you for your comments, the data of *K. michiganensis* BD177 genome was referenced in publicly accessible database 'NCBI' with Accession ID: PRJNA602959. We have changed this part in the revised version in lines **386-393** as follows;

“However, our recent genome wide sequencing and phylogenetic analysis showed that the *Klebsiella* sp. BD177, which was isolated from the *B. dorsalis* gut and previously identified as *K. oxytoca* on the basis of 16S RNA sequencing [32], has 97.74% ANIm with the reference genome of *K. michiganensis* DSM 25444 (=W14) (Accession: PRJNA388837) but only 92.20% ANIm with the *K. oxytoca* ATCC 13182 reference genome; thus, it had been classified as *K. michiganensis* BD177 (Accession: PRJNA602959), which was separated and independent from *K. oxytoca* according to average nucleotide identity (ANI), a genomic gold standard for bacteria species identification [42].”

**Major Issues: Key Experiments Required for Acceptance**
**Reviewer #1:** na

**Reviewer #2:**

Past studies with *D. melanogaster* and *D. suzukii* have shown that microbivorism can enhance host health and development. These effects can be achieved by live microbes, or by dead microbes that are given to the host continually and at levels that effectively mimic the propagation and continual presence of live microbial growth. In Bing et al. (2018), live microbes enhance *D. suzukii* development, and heat-killed microbes also rescue *D. suzukii* development in a dose-dependent manner. Similarly, in Yamada et al. (2015) and Keebaugh et al. (2018), the lifespan-extending effect of microbes can be fully mimicked by dead microbes that are continually given to flies at a level that is equivalent to what an initial inoculum of live microbial growth would have produced. Essentially, microbes that grow better are better at rescuing host health under nutritional stress. This nutritional aspect of microbes should therefore be tested as a potential factor in host–microbe studies, especially when using hosts that naturally feed on food substrates that contain microbial growth, like fruit and vinegar flies.

The valuable suggestions of reviewer were taken into account and we agree with the reviewer’s remarks that such potential factor in host–microbe studies, e.g., nutritional aspects of dead microbes that are continually given throughout the life and their effects on survival-extension cannot be ruled out as shown by recent studies of Yamada et al. (2015), Bing et al. (2018) and Keebaugh et al. (2018). Following these interesting guidelines, we performed a series of experiments using equivalent or increased and recurring supply of dead microbes and found interesting results that helped to expand the scope and significance of our study. The results of revised experiments have been added in the lines ‘**162-171**’ in new version as follows;

“A single dose reinfection or recurring supply with heat-killed (HK) *K. michiganensis* equivalent to the levels of live *K. michiganensis* (10^8^ CFU/ml) showed no significant effect on the survival time of ABX flies (Fig 3A, *P*>0.05) or in another independent longevity test (Fig 3B, *P*>0.05) compared with ABX control postexposure to 10°C. Moreover, increasing the quantity of HK *K. michiganensis* (10^9^ CFU/ml) given to ABX flies throughout life did not even rescued the median lifespan (5.5 days) over the axenic control (5 days), and to a significantly lesser extent (*P <* 0.0001) than that of live *K. michiganensis* inoculation (Fig 3A). Taken together, our results show that survival of flies cannot be mimicked by HK *K. michiganensis*, suggesting that microbes may stimulate indirect processes or host metabolism, rather than directly providing nutritional benefits to the host during low-temperature stress.”

Data within this paper support the idea that *K. michiganensis* grows well in the cold because it reaches CFU levels equivalent to the total microbial load of conventional *B. dorsalis*. This suggests that microbial growth alone could be the main factor in how microbes influence cold survival. One way to test this hypothesis might be to provide each live microbe at equivalent initial doses, and then monitor the growth of each microbe over time to determine if host benefits are specific to *K. michiganensis*, or if general microbial growth can stimulate host metabolism in a way that is beneficial during cold stress. Further, the authors should properly test if heat-killed K. michiganensis can mimic live *K. michiganensis* in survival trials. Heat-killed K. michiganensis should be continually given to the flies at levels equivalent to live microbial growth in order to determine whether live microbes actively influence the host in a way that boosts cold survival. Directly quantifying bacterial growth rates at different temperatures might also be informative.

Thank you for the interesting comments. To investigate the dynamics of *K. michiganensis* during low temperature, we added an experiment to examine CFU levels of gut bacteria at 0 dpe (10 days posteclosion or before exposure to 10°C), and compared with CFU levels at 5 and 10 dpe to 10°C, after the reinfection of ABX flies with live *K. michiganensis* for 5 days at normal room temperature. The results showed that feeding of live *K. michiganensis* gave 2.087 x 10^7^ ± 1.545 x 10^6^ CFUs gut^-1^ (mean ± SE of 10 individual flies), which is nearly equivalent to the levels found in conventional flies 2.36 x 10^7^ ± 1.626 x 10^6^ CFUs gut^-1^ (mean ± SE of 10 individual flies), at 0 dpe to 10°C. Notably, levels of CFUs after feeding live *K. michiganensis* at 0 dpe to 10°C (2.087 x 10^7^ ± 1.545 x 10^6^ CFUs gut^-1^) is also nearly equivalent to the levels of live *K. michiganensis*-reinfected flies (1.97 x 10^7^ ± 1.87 x 10^6^ CFUs gut^-1^) at 5 dpe to 10°C, or at 10 dpe to 10°C (1.937 x 10^7^ ± 3.014 x 10^6^ CFUs gut^-1^) (*P*>0.05) (see following Fig. 1). These results suggest that ABX flies fed with live *K. michiganensis* for 5 days (at 0 dpe to 10°C) at normal room temperature is sufficient for *K. michiganensis* to recolonize and recover to the normal CFU levels, and can be sustained or maintained in the gut environment even after 5 and 10 dpe to 10°C to the levels of conventional flies gut that, in turn, promote host resistance ability to low-temperature stress.

On the other hand, after an overnight culture of *K. michiganensis* (at an OD of 2)*,* different powers of serial dilutions, e.g., 10^-5^ and 10^-7^ were made, and 100 μl of each dilution was inoculated in triplicate via the spread plate method on Luria-Bertani (LB) solid media under aerobic conditions. Then, plates were placed at 10°C for incubation, and investigated *K. michiganensis* growth for 24 h to 72 h, unfortunately, we did not observe the growth of *K. michiganensis* directly at 10°C. Taken together, those results suggest that *K. michiganensis* can only survive in the gut environment during cold stress treatment.

**CFU/gut**

**Fig. 1. Microbial load in gut homogenates is maintained at different ages after feeding live *K.*** ***michiganensis* to ABX flies at 0, 5 and 10 dpe to 10°C*.*** Colony-forming units (CFUs) of flies (mean ± SE of 10 individual fly guts); the average number of cultivated microbial communities resulting from CFU in ABX control fed with live *K. michiganensis* after 0 dpe to 10°C gave 2.087 x 10^7^ ± 1.545 x 10^6^ CFUs gut^-1^ vs. that of conventional flies, which showed 2.36 x 10^7^ ± 1.626 x 10^6^ CFUs gut^-1^ (*P*>0.05) (mean ± SE of 10 individual flies). Additionally, CFU levels after feeding live *K. michiganensis* at 0 dpe to 10°C (2.087 x 10^7^ ± 1.545 x 10^6^ CFUs gut^-1^) is also nearly equivalent to the levels of live *K. michiganensis*-reinfected flies at 5 dpe to 10°C (1.97 x 10^7^ ± 1.87 x 10^6^ CFUs gut^-1^), and 10 dpe to 10°C (1.937 x 10^7^ ± 3.014 x 10^6^ CFUs gut^-1^) (*P*>0.05). CFU data of conventional and live *K. michiganensis*-reinfected flies were compared using ANOVA followed by Tukey test and *P*>0.05 denoted as nonsignificant (ns). The error bars indicate standard error of the mean (SEM). CFU represents the mean ± SE of 10 individual flies (n=3).

Next, in accordance with the results of initial survival trials and reviewer suggestion, we next asked whether host benefits are specific to *K. michiganensis* or if general microbial growth can stimulate host metabolism in a way that is beneficial during cold stress. To test this hypothesis, we reinfected axenic flies with each live gut microbe at an equivalent initial dose, and then we monitored the growth of each microbe (CFUs) 5 dpe and 10 dpe to 10°C. Interestingly, we found that that only *K. michiganensis* can form a sustained colony in the ABX flies gut equivalent to the levels found in conventional flies and/or significantly higher than that of all other tested strain, suggesting that *K. michiganensis* microbial quantity is influential to promote host resistance ability to low-temperature stress (**S2 Fig B and C**). Therefore, we concluded that the host benefits under temperature stress are specific to *K. michiganensis*. The results of these experiments have been added in the revised draft in the lines **‘175-184’**. Similarly, to test whether other gut bacterial strains could grow directly at 10°C, we investigated the CFU levels of each gut bacterial strain via the spread plate method on Luria-Bertani (LB) solid media and incubated at 10°C for 24 h to 72 h. Again, we did not observe any bacterial growth directly at 10°C, suggesting that bacteria can only survive in the gut environment during cold stress treatment.

The answer of question “if heat-killed *K. michiganensis* can mimic live *K. michiganensis* in survival trials” have been incorporated in the above paragraph in the lines **89-98**.

Although the manuscript focuses on the "gut microbiota," the data doesn't convincingly rule out contributions from other sources. I assume that the axenic treatment doesn't simply remove the gut bacteria, but also eliminates it from all niches (on the fly, in the fly, and in the fly environment/food). How does one show that the causal factor is the gut microbiome in particular? Do these flies have a well-established, stable gut microbiome?

We agree with the reviewer’s comment that axenic treatment is a major challenge for the repeatability in gut microbiota studies. It might be possible that axenic treatment could eliminate bacteria from all niches, however, it is inevitable that the background of gut microbiota composition from our work is distinct from other studies. Additionally, after 10 days post eclosion all the testing flies were shifted to the separate growth chamber at 10°C (free of antibiotic artefacts), where they were provided with sterile liquid diet or HK gut bacteria (HK microbes only for recurring experiments). Moreover, the effect of gut bacteria reinfection via feeding each gut microbe to ABX flies individually was further confirmed by survival trials, which indicated that resistance to low-temperature stress was specific to the gut bacteria, e.g., *K. michiganensis*, and not from other niches or other bacteria (**S2 Fig A**).

Additionally, according to our previous studies, by combining microbial culture dependent and -independent techniques we found that *B. dorsalis* harbour a stable bacterial community in which dominant members belong to Enterobacteriaceae with major species of *Klebsiella*, *Citrobacter*, *Enterobacter* (Wang et al., J Appl Microbiol 2011; Yao et al., ISME J. 2016; Cai et al., Evol Appl. 2018).

**Reviewer #3: Major revisions:**

1)Arginine and proline pathways are dependent on the TCA cycle and a previous study has linked mitochondrial activity to the arginine and proline induction in *Drosophila*'s resistance to low temperature ("Physiological basis for low-temperature survival and storage of quiescent larvae of the fruit fly *Drosophila melanogaster* "Koštá et al. Sci. Rep 2016), I believe that mitochondria status should be assessed in this fly by transmitted electron microscopy or mitotracker staining followed by fluorescent microscopy and measurement of mitochondria morphology to determine whether the metabolic changes could be linked to the mitochondria activity. Thus mitochondria status should be tested on conventional, axenic and *K. michiganensis* recolonized flies to assess whether the bacterium affect mitochondria activity in general. Moreover, to measure the mitochondria functionality and also to better define the metabolic mechanisms that promote the host survival, ATP measurements should be taken in all conditions.

We really appreciate for such valuable comments of honorable reviewer to expand the scope of our research. We have assessed the mitochondria status in conventional, ABX (axenic) and *K. michiganensis* recolonized flies gut tissues by transmitted electron microscopy (TEM) and also measured the ATP levels. We represented the text in lines **‘334-357’** under a single title “Gut bacteria maintains mitochondrial morphology and ATP levels during low-temperature stress”, as follows;

**“Gut bacteria maintains mitochondrial morphology and ATP levels during low**-**temperature stress**

To investigate a link between gut bacteria and mitochondria activity for the arginine and proline induction to promote host resistance during low-temperature stress, we observed gut tissues of conventional, *K. michiganensis*-reinfected or ABX flies 5 dpe to 10°C, through transmission electron microscopy (TEM). The results showed that in the absence of gut bacteria (ABX flies), low-temperature stress caused severe destruction in cristae structure and thus resulted in abnormal circular shapes of mitochondria compared with conventional flies (Fig 9 A- C). On the other hand, *K. michiganensis* inoculation helped the host to maintain mitochondrial morphology, clearly different from that of ABX flies following exposure to low-temperature stress. Furthermore, mitochondrial morphology of ABX flies were severely distorted compared with conventional or *K. michiganensis* inoculated flies (Fig 9 D; percentage of distorted mitochondria in Conventional: 5.027 ± 0.9992%; *K. michiganensis*: 14.95 ± 1.709%; ABX flies: 77.99 ± 1.838%, *P*<0.0001). Number of cristae in ABX flies were also significantly reduced by 71 % than that of *K. michiganensis* fed flies (Fig 9 E). These experimental observations led us to conclude that, once inoculated, *K. michiganensis* might help the ABX flies to maintain a dynamic equilibrium in mitochondria and improves its metabolic functions that might induce arginine and proline to the levels sufficient to promote host resistance to low-temperature stress.

Moreover, to define the mitochondria functionality and metabolic mechanisms that promote host survival during cold stress, we measured the amount of ATP in the gut tissues of these flies. We found that ATP concentration was significantly reduced by 78.64% in the gut tissues of ABX flies compared with the conventional flies 5 dpe to 10°C (Fig 9 F, *P*<0.05). However, after the feeding of live *K. michiganensis* to ABX flies, ATP concentration in the gut tissues was reestablished and significantly increased by 377% compared with those in the ABX flies (Fig 9 F).”

Additionally, we have added methods for TEM, ATP levels and damage percentage in the revised draft the lines ‘**681-691**’ and ‘**614-619**’, respectively.

2) The authors observed that high TG in the hemolymph is a metabolic features of conventional resistant flies to the temperature stress. The level of TG or TG storage should be assessed in the fly fat body to understand whether fat synthesis or storage consumptions are also responsible for the survival mechanisms.

We are thankful for the reviewer’s suggestion. The experiment was performed and results have been incorporated in the revised version in the lines ‘**328-333**’, as follows;

“To understand whether fat synthesis or storage consumptions are also responsible for the survival mechanisms, TG levels were assessed in the fly fat body tissue among conventional, *K. michiganensis*-reinfected or ABX treatments. However, we did not find significant differences in the TG levels among those different treatments (*P*>0.05, S4 Fig C), indicating that fatty acid molecules might not be involved in the host resistance to cold stress.”

Moreover, we have added methods to measure the TG levels in the fly fat body tissues across all treatments, in the revised draft the lines ‘**596-600**’.

3) Are lipid synthetic genes or catabolic genes up-regulated in the transcriptomic analysis to justify this TG increase? My apologies if this was not clear to me.

We really hold up a higher regard for reviewer’s logical question. In our transcriptomic data, we found some lipid synthetic genes significantly enriched, e.g., fatty acid synthase (Cluster-10843.22928), lipase member H-A (Cluster-10843.23140), phospholipid scramblase 2-like (Cluster-10843.14138), inositol-3-phosphate synthase (Cluster-10843.30885), acyl-CoA Delta desaturase-like (Cluster-10843.33193), CDP-diacylglycerol--inositol 3-phosphatidyltransferase (Cluster-10843.44041) etc., which might be involved in this TG increase in conventional flies hemolymph than that of axenic (ABX) control. But, in transcriptomic analysis, fatty acid metabolisms, Biosynthesis of unsaturated fatty acids, Glycerophospholipid metabolism, Glycerolipid metabolism pathways etc. were not significantly enriched in *K. michiganensis*-reinfected vs. axenic (ABX treated) flies or conventional vs. axenic (ABX treated) flies comparison. Although, the increase of TG levels was observed in conventional flies’ hemolymph but in transcriptomic analysis we found non-significant enrichment of lipid metabolism pathways, which might be possible due to different methodologies. On the other hand, we find and focused on a commonly present pathway “Arginine and proline metabolism pathway” genes and molecules significantly up-regulated in the above mentioned comparisons, which we have further confirmed via qPCR, RNAi, and microinjections experiments.

Initially, we suspected that lipid genes could be responsible to cold stress resistance of host and we tested the relative expression of some genes, e.g., the relative expression levels of fatty acid synthase (FASN) gene, a key enzyme that catalyzes the reductive synthesis of long-chain fatty acids (which were also up-regulated in metabolomics analysis of *K. michiganensis*-reinfected vs. axenic (ABX treated) flies, e.g., arachidonic acid, linoleic acid) at 0 day and 5 dpe to 10°C in all those treatments. But, again we did not obtain any significant results among different treatments. Additionally, our microinjection experiments of other fatty acid molecules that were up-regulated in *K. michiganensis*-reinfected vs. axenic metabolomics analysis further confirmed that fatty acid molecules/enzymes might not be involved in the promotion of host resistance to cold.

4) Injection of flies with linoleic acid and arachidonic acid does not necessarily recapitulate the metabolic phenotype linked to temperature stress resistance. TG are made of different saturated and unsaturated fatty acid chains. What kind of TG are in the hemolymph? Because the exact types should be injected to rule out whether TG in the hemolymph are responsible or not for the survival. A diet on holidic media supplied with different fatty acid chains should be given to the flies in different conditions before coming to the conclusion that TG have no impact on the low-temperature resilience of the host.

We agree with the reviewer’s comments that TG are made up of different saturated and unsaturated fatty acid chains. But, unsaturated fatty acid molecules were not significantly enriched in the hemolymph except Ricinoleic acid, which was down-regulated in *K. michiganensis*-reinfected vs. axenic metabolomics comparison. However, other types of fatty acid molecules being up-regulated in our data (presented categorically in table below), e.g., Geraniol, Nonadecylic acid, 6-Keto-decanoylcarnitine, 3-Hydroxy-9-hexadecenoylcarnitine, 3-Hydroxy-9-hexadecenoylcarnitine, Latanoprost, Heptadecanal (Rhawn-Shanghai, China), Dodecanoic acid and Ricinoleic acid (Yuanye-Shanghai, China), were tested via microinjection into the ABX treated flies and subjected to low-temperature stress treatment. But, we did not find any significant effects on the survival of ABX flies, indicating that fatty acid molecules/enzymes might not be involved in the host resistance to cold. The results of microinjection experiments have been incorporated in the **‘S4 figure A’** in the revised version.

| **Metabolites** | **Category** |
| --- | --- |
| Geraniol | Fatty acyl |
| 6-Keto-decanoylcarnitine | Fatty acid ester |
| Dodecanoic acid | Saturated fatty acids |
| Nonadecylic acid | Saturated fatty acids |
| 3-Hydroxy-9-hexadecenoylcarnitine | Fatty acid ester |
| Latanoprost | Fatty acid ester |
| Heptadecanal | Fatty acyl |
| Ricinoleic acid | Unsaturated fatty acid |
|  |  |

5) Is this bacterium important to provide resistance to other types of stress? Other stressing should be tested to verify whether it specifically helps the host to activate temperature stress response pathways or also control responses to other common stresses such as UV exposure.

We are thankful for the reviewer’s interesting query. We have tested conventional flies as well as *K. michiganensis* response against UV stress treatment. The median survival time for conventional and ABX flies fed with *K. michiganensis* were 20.50 and 15 days, respectively, compared with 18.50 days found in the axenic flies (S5 Fig). These results showed that there were non-significant differences in the median survival time among different treatments, suggesting that gut bacteria e.g., *K. michiganensis* might not have symbiotic effect to provide resistance against UV stress. The methods and results are presented in the revised version in lines ‘**601-613**’ and **S5 figure**, respectively.

6) Does injection with arginine and proline together improve the viability?

The logical comments of reviewer helped us to understand clearly about the synergistic effects of these important metabolites. We got encouraging results in survival assays, after injecting L-arginine + L-proline to the ABX treated flies, the median survival time was improved than that of their individual applications. The results have been modified in the revised version in lines ‘**322-326**’ and ‘**figure 8**’ as follows;

“Furthermore, we evaluated the synergistic application of L-arginine + L-proline to the ABX flies’ survival. The results showed that after injecting the combination of L-arginine + L-proline, the median survival time of ABX flies was considerably increased by 150% (15 days) compared with the ABX control (Fig 8), suggesting the synergistic potential of these amino compounds under cold stress.”

7) And does ds RNA-mediated silencing of target genes that affect both pathways reduce the survival?

We are again thankful for the reviewer suggestion that guided us to verify our above results via RNAi. After performing these experiments we have added a new section entitled “Combined effect of key genes silencing on the survival of conventional flies postexposure to low-temperature stress ” in the revised draft in lines ‘**303-313**’ and ‘**figure 7 F and G**’ as follows;

**“Combined effect of key genes silencing on the survival of conventional flies postexposure to low-temperature stress**

According to the previous experiments, we found that two key genes Pro-C and ASS silencing considerably reduced the median survivals of flies (Fig 7 E). Next, we made the combination of dsPro-C and dsASS to silence these genes and evaluated the synergistic effect on the survival of conventional flies under low-temperature stress. The results showed that injection of dsPro-C + dsASS significantly decreased the Pro-C and ASS mRNA levels in conventional flies (Fig 7 F), and as a result their median survival was markedly reduced by 67% postexposure to low-temperature stress of 10°C (Fig 7 G). These results suggest that a combined decrease in Pro-C and ASS mRNA levels might cause a highly significant reduction in the survival time of flies during low-temperature stress.”

**Minor Issues: Editorial and Data Presentation Modifications**
Please use this section for editorial suggestions as well as relatively minor modifications of existing data that would enhance clarity.

**Reviewer #1:** The ms., is very well written and despite the complexity, easy to follow. There are a few very minor points that need attention:
Line 73- “The mechanisms underlying cold acclimation responses are the main focus of much research in insect species”. References are needed to back this up. E.g.:
Overgaard, J., MacMillan, H.A., 2017. The Integrative Physiology of Insect Chill Tolerance, Annual Review of Physiology, Vol 79, pp. 187-208.
Teets, N.M., Denlinger, D.L., 2013. Physiological mechanisms of seasonal and rapid cold-hardening in insects. Physiological Entomology 38, 105-116.
Thanks for the comment. The references have been added in the revised version in text and references parts of revised draft.
110- [31]
We have modified the change in the revised version.

112- figure 2- but no reference to figure 1? Use fig 1 in the methods section and renumber the figures accordingly.

We are thankful for the reviewer’s precise observation. We have rearranged all figure numbers throughout the manuscript, accordingly.

121- figure 3- extend the x axis to show the full extent of survival (as in the other survival figures), not just the first 6 days, or explain why not.

Thanks a lot for the suggestion. We have extended the x axis to show the full extent of survival in the modified “figure 2” in the revised draft.

**Reviewer #2: Other suggestions:**

The fly diet should be described in better detail.

The diet we used in our experiments has been described in the revised draft under “Fly culture” and “Reinfection of ABX flies with gut symbionts” sections.

The heat-killed dosing protocol should be described in better detail.

The heat killed dosing protocol (10^8^ and 10^9^ CFU/ml) has been described in better detail in the revised version in the lines ‘**575-581**’ “Reinfection of ABX flies with gut symbionts” section.

The authors show that oral antibiotics do not influence host survival under normal rearing conditions, but what about treatment conditions (in the cold)?

In our initial trials, we performed the survival trials successfully at room temperature (Fig s1 A) using axenic (ABX) and conventional flies, in order to make sure that antibiotic artefacts were not present in axenic (ABX) flies. But, when we did independent survival and longevity assays under low-temperature (10°C), the survival of axenic (ABX) flies were significantly reduced and recovered after the reinfection of gut bacteria *K. michiganensis* following exposure to low-temperature (10°C), as shown in the “**figure 3 A and B**”, indicating that under cold treatment conditions survival time of axenic (ABX) flies is decreased due to the absence of bacteria and not by the antibiotic treatment. In other words, gut bacteria is the main factor that helps the host to promote resistance under cold conditions.

The reinfection protocol is not normalized by bacterial load (CFUs) because a general OD measure was used. Thus, *B. dorsalis* were likely given very different levels of microbes at the start of monoculture experiments. If microbial growth alone is influential on cold survival, then different inoculum sizes could influence microbial growth dynamics and ultimately host survival. These experiments should be done in a controlled manner in which the initial CFU is normalized across microbial strains.

We appreciate the kind remarks and regret for unclear explanation in the initial submitted version. However, we have explained clearly in the revised file that at the start of monoculture experiments reinfection protocol was normalized by bacterial load (CFUs). The detailed modifications have been made in the lines ‘**561-585**’ of revised draft as follows;

**“Reinfection of ABX flies with gut symbionts**

In trial 2 fitness tests, to acquire the key bacterium among the all isolated strains, we reinfected the ABX flies with all 15 isolated strains (S2 Table)*.* For monoculture experiments, equivalent levels of each microbe were introduced to ABX flies in order to ensure their potential beneficial effects (if any) on the survival of flies under low-temperature stress. The inoculum was prepared as follows: Optical density (OD) was measured on an overnight culture of each bacterial species used. Then, 10^-5^ and 10^-7^ powers of serial dilution of the bacterial cultures were made and 100 μl dilutions were inoculated on Luria-Bertani (LB) solid media using the spread plate method and incubated under aerobic conditions at 30°C for 24 h. Next, we determined CFUs on each plate resulting from the different bacterial cultures, individually. The resuspension volume was determined based on the empirically determined constants (CFU ml^-1^ at an OD of 2) to make an equal (normalized) dose of 10^8^ CFU/ml [46] for all tested strains. The cells from each bacterial cultures were pelleted at 3600 X g for 15 minutes and resuspended in the liquid diet mixture of 2.5% yeast extract, 7.5% sugar, 2.5% honey and 87.5% H_2_O in equal parts and fed to the ABX flies for 5 days (Fig 10). HK gut bacterium (*K. michiganensis*) was prepared by autoclaving at 121°C for 30 minutes, and aliquots were stored at -80°C. A dose of 10^8^ CFU/ml HK *K. michiganensis* was provided to the ABX flies for 5 days (denoted as “single dose”). Flies reinfected with live or HK microbes were then supplied with sterile liquid diet till the end of survival or longevity assays. Recurring supply of HK *K. michiganensis* to an initial dose of 10^8^ CFU/ml (equivalent to the initial inoculum of live *K. michiganensis*) or 10^9^ CFU/ml (increased dose) were supplied ad libitum till the end of survival or longevity assays. Conventionally reared adult flies were fed with sterile liquid diet throughout the experiment. We generally marked '0 days' before exposure to 10°C, which was 10 days posteclosion, for all studies. Fifteen days post-eclosion or exposure to normal rearing temperature of 28°C was also considered as 5 dpe, in the whole study to ensure the usage of same-aged flies (Fig 10).”

There were CFUs detected in flies that were supposed to be axenic or given heat-killed microbes. Was this contamination? Unless there is an explanation for this, the presence of live microbes is a confounding factor in all experiments using flies that should be absent of live microbial growth.

We are thankful for the kind remarks. The gut microbiota composition of *B. dorsalis* is very complex, and even multiple combinations of antibiotics are unable to deplete the gut bacteria completely or it might be possible that antibiotic resistant bacterial strains can regrow after stopping the antibiotics feed. However, in our whole study we feed antibiotic orally to the flies only for 5 days and then antibiotic treated flies were shifted to the other diets containing bacteria (for recolonization studies) OR to a fresh and autoclaved liquid diet at every food change to avoid any expected contamination (the details are shown in schematic representation in figure 10). However, in our study we found that antibiotic treatment significantly depleted (>95%) the gut bacterial population than that of recolonized flies or conventional flies, which was the major purpose of this study.

However, we changed the word "**axenic**" with “**ABX**” (ABX stands for antibiotic treated flies) throughout the text, figures and supplementary datas. Besides, we find some bacterial populations in the single dose heat-killed *K. michiganensis* treatment in our qPCR analysis too, which might be due to the high detection sensitivity of inactive bacterial components via qPCR technique, but this population was still significantly less than that of recolonized flies or conventional flies. Further, survival assays supported our results too that a negligible amount of gut bacteria detected in single dose dead bacteria fed or antibiotic treated flies does not improve the survival compared with live *K. michiganensis* treatment or conventional flies.

**Reviewer #3: Minor issues:**

1) Figure made of one or two panels could be combined in single figure to facilitate the reader.

We are thankful for the kind remarks. In revised file, each figure has been modified accordingly.
2) The experimental details for the metabolomic analysis should be provided (e.g. how many ul of hemolymph, where they normalized to number of flies? weight? protein of the carcasse?) as well as for the transcriptomic (amount of RNA, kit used, seq platform etc.).

Our apologies if it was not clear. However the details of metabolomic analysis have been added in the revised draft in lines ‘**620-630**’. For transcriptomic analysis, informations of amount of RNA, kit used or sequence platform etc. have been added in the revised file in the lines ‘**640-644**’ , however there were lot more details of transcriptomic analysis therefore other details have been given in supplementary informations in “S1 Methods”.

New **References** added to the re-submitted draft

18.	Overgaard J, MacMillan HA. The integrative physiology of insect chill tolerance. Annu Rev Physiol. 2017; 79: 187-208. <https://doi.org/10.1146/annurev-physiol-022516-034142>.

19.	Teets NM, Denlinger DL. Physiological mechanisms of seasonal and rapid cold‐hardening in insects. Physiol Entomol. 2013; 38: 105-116. https://doi.org/10.1111/phen.12019.

44.	Bing X, Gerlach J, Loeb G, Buchon N. Nutrient-dependent impact of microbes on *Drosophila suzukii* development. MBio. 2018; 9: e02199-02117.

45.	Keebaugh ES, Yamada R, Obadia B, Ludington WB, William WJ. Microbial quantity impacts *Drosophila* nutrition, development, and lifespan. iScience. 2018; 4: 247-259. https://doi.org/10.1016/j.isci.2018.06.004.

52.	Shutt TE, McBride HM. Staying cool in difficult times: mitochondrial dynamics, quality control and the stress response. Biochim Biophys Acta. 2013; 1833: 417–424. https://doi: 10.1016/j.bbamcr.2012.05.024.

53.	Paumard P, Vaillier J, Coulary B, Schaeffer J, Soubannier V. The ATP synthase is involved in generating mitochondrial cristae morphology. EMBO J. 2002; 21: 221-230. <https://doi.org/10.1093/emboj/21.3.221>.

54.	Cogliati S, Frezza C, Soriano ME, Varanita T, Quintana-Cabrera R. Mitochondrial cristae shape determines respiratory chain supercomplexes assembly and respiratory efficiency. Cell. 2013; 155: 160-171. https://doi.org/10.1016/j.cell.2013.08.032.

55.	Harner M, Korner C, Walther D, Mokranjac D, Kaesmacher J, Welsch U, Griffith J, Mann M, Reggiori F, Neupert W. The mitochondrial contact site complex, a determinant of mitochondrial architecture. EMBO J. 2011; 30: 4356-4370. <https://doi.org/10.1038/emboj.2011.379>.
