# Supplementary material for: Gut microbiota promotes host resistance to low-temperature stress by stimulating its arginine and proline metabolism pathway in adult Bactrocera dorsalis
Source: PLoS Pathog. 2020 Apr 15;16(4):e1008441. doi: 10.1371/journal.ppat.1008441 (PMC7185725; doi:10.1371/journal.ppat.1008441)
Supplement: Attachment — Submitted filename: Response letter minor revisions.doc [file ppat.1008441.s012.doc]

**Institute of Urban and Horticultural Entomology**

**College of Plant Science and Technology**

**Wuhan 430070, Hubei**

**P. R. China**

**Tel: 0086-27-8728-6962**

**Fax: 0086-27-8728-0796**

**华 中 农 业 大 学**

**Huazhong Agricultural University**

February 24, 2020

To The Editor

PLoS Pathogens

Dear Sir/Madam,

Thank you for your mail concerning our manuscript entitled “**Gut Microbiota Promotes Host Resistance to Low-Temperature Stress by Stimulating its Arginine and Proline Metabolism Pathway in Adult *Bactrocera dorsalis***”, for consideration to **PLoS Pathogens.**

The remarks of both reviewers and editors were taken into account and were discussed extensively one by one with the co-authors. We have proofed and revised the manuscript carefully. All the minor comments have been considered carefully and corrected according to the reviewer’s suggestion.

In accordance with reviewer’s remarks, the manuscript has now been extensively revised to conform to guidelines for publication in **PLoS Pathogens**. It has also been revised again by the team at American journal experts (AJE) for correct English usage.

Our answers (in red) to **each comment** of the reviewers can be found in the following pages and revised draft.

We do hope that you will now find our revised manuscript suitable for publication in PLoS Pathogens journal in its present form.

Thanking you in anticipation of favorably considering our manuscript.

Yours sincerely,

Hongyu Zhang Ph.D. Professor (*On the behalf of all authors*)

**Minor Issues: Editorial and Data Presentation Modifications**

**Reviewer #1:** na

**Reviewer #2:**

The authors have added additional data that bolster the conclusions. The new data are substantial, and while other studies could be added to further support the findings (i.e. additional controls), I don't think they're absolutely necessary. The authors should still consider conducting these studies or acknowledging them by modification of the text.

1) Antibiotic treatment has been shown to affect mitochondrial function in various animals. Hence, although the authors' new data on mitochondria morphology is consistent with their overall interpretations, it might be valuable to repeat key studies using a truly axenic protocol (without using antibiotics). I'm not sure that's possible with their system. Nonetheless, reinfection with *K. michiganensis* rescues the effect, so I don't think this is a huge concern.

We agree with the reviewer’s interesting comment that antibiotic treatment has been shown to affect mitochondrial function in various animals in other studies. It could be valuable to conduct key studies using a truly axenic protocol (without using antibiotics), unfortunately, we do not have sufficient experimental techniques to maintain *B. dorsalis* germ-free adults, like mice or other flies, e.g., germ-free strains of *Drosophila*. However, in view of this important suggestion, we have discussed and modified the text in the revised version in the lines **477-485**, as follows;

“Several antibiotics have been reported to be toxic to mitochondria in other animals [56]. It might be valuable to conduct cold stress studies using a truly axenic or germ-free flies, without using antibiotic treatments. We were unable to perform a low-temperature challenge on truly axenic or germ-free flies, however, our study still provides the clear effects of *K. michiganensis* reinfection that rescues the survival of ABX flies and helps the host to maintain mitochondrial morphology at low-temperature stress, suggesting that the reduced median survival time of ABX flies, which were generated via treatment with oral antibiotics cocktails as most researchers did in this research field [33], at low- temperature resulted from elimination of the gut microbiota, not from their toxic effects to mitochondria.”

2) The authors could also consider looking at mitochondria after amino acid injection (and/or dsRNA manipulations). This would greatly strengthen the findings.

We are thankful for the reviewer’s suggestion. This is a novel idea to investigate the mitochondria morphology after highly up-regulated amino acid injection, e.g., arginine and proline and/or dsRNA manipulations. In the current study, we only focused on the gut bacteria ability to maintain healthy mitochondria during cold stress. However, the connection between amino acids injection and dsRNA manipulations on the mitochondrial morphology cannot be ruled out during low-temperature stress and should be studied in the future. In view of these important points, we have modified the text in the revised version in the lines ‘**475-477**’, as follows;

“It might also be interesting to investigate the mitochondrial morphology after the injection of highly expressed amino acids, e.g., L-proline and L-arginine and/or by silencing their corresponding genes in the future.”

3) From the types of studies that were done, it's hard to say whether injection of proline and arginine together results in an additive or synergistic effect. This doesn't have to be determined here, but could be discussed. I may have missed it, but did the authors also test other amino acids, as another control on the specificity for these amino acid pathways? I think that experiment would be a really nice control to have, but I don't think it's absolutely needed.

We really appreciate the comments of honorable reviewer. In previous major revisions, reviewer#3 asked us to evaluate the synergistic effect of arginine and proline together, therefore, we performed the microinjection experiment of these amino compounds that resulted in the increased survival time of ABX flies under cold stress. Our apologies if it was not clear, however, we did not test other amino acids, but focused on these two amino compounds (arginine and proline) from this commonly present pathway “Arginine and proline metabolism pathway” because of their consistence and significant up-regulation in both metabolomics and transcriptomic analysis. Furthermore, we confirmed their corresponding genes function via qPCR and RNAi technique. We have discussed all these findings in the earlier draft, which are presented in the lines **262-333** in the revised version.

4) The use of heat-killed bacteria greatly helps support the authors' conclusions. The maximum provision of heat-killed bacteria was 10^9 at each food change. This is compared to the single inoculation of live bacteria of 10^8. My question here is, with single live inoculations, how much environmental bacterial growth occurs and is the fly constantly receiving reinoculations from live bacteria from the environment? If so, are the bacterial CFUs that the hosts are exposed to equivalent to the 10^9 at each food change? From my calculations from other publications, it seems that bacterial growth rates can sometimes require repeated exposures of 100-fold more dead bacteria to match the rapid growth rates of live bacteria. The authors should be quite clear that they are using roughly equivalent amounts to truly justify their interpretations.

We really appreciate for such valuable comments of reviewer. In our study, the dose of live *K. michiganensis* (10^8^ CFU/ml) that resulted in maximum survival extension, produced 1.97 x 10^7^ CFUs (S2 Fig. B) associated with the fly gut after 5 dpe to 10°C, which is equal to the 3.94 x 10^6^ CFUs/day. While, the maximum provision of heat-killed *K. michiganensis* was 10^9^ CFU/ml at every food change with 2 days interval, which is equal to the 5 x 10^8^ CFUs/day. According to these calculations, dosage of heat-killed *K. michiganensis* (5 x 10^8^ CFUs) per day was 126- fold higher than that of live *K. michiganensis* (3.94 x 10^6^ CFUs) per day. In addition, we agree with the findings of previous publications as well as reviewer’s point of view that bacterial growth rates can sometimes require repeated exposures of 100-fold more heat- killed bacteria to match the rapid growth rates of live bacteria. In the current study, we provide a very high dosage of heat- killed bacteria (>100-fold) to match the rapid growth rates of live bacteria and to justify the interpretations of our data.

The number of microbes present in the fly environment results from multiple factors including microbial growth rate on the food surface, ingestion by flies, and spreading through fly excretion and external contact. These factors are likely further complicated by parameters such as the number of flies, enclosure size, and food surface area. However, the fly gut microbiota is generally transient under laboratory conditions (Blum et al., mBIO 2013) and a recent study showed that the number of live microbes in the fly gut was found to be likely reflective of the quantity in the fly environment (Keebaugh et al., iScience 2018). To the best of our knowledge, available data on the microbiota of *B. dorsalis* are unfortunately limited, which restricts understanding that how much live bacteria does fly constantly receives from the environment, which should be studied in future.

Reviewer #3: I still believe that the Figures could be filled more since most of them (e.g. Figure 1 and 2) still comprise only 2 panels and it is awkward.

We regret for the negligence. In the revised draft we have merged figure 1 and 2- comprising of more than 2 panels. While, other figures have not been changed because those were being presented according to the type of their specific experiments. The figure numbers have been modified in the revised draft.

Figure Files:

While revising your submission, please upload your figure files to the Preflight Analysis and Conversion Engine (PACE) digital diagnostic tool, [https://pacev2.apexcovantage.com](https://pacev2.apexcovantage.com/). PACE helps ensure that figures meet PLOS requirements. To use PACE, you must first register as a user. Then, login and navigate to the UPLOAD tab, where you will find detailed instructions on how to use the tool. If you encounter any issues or have any questions when using PACE, please email us at [figures@plos.org](mailto:figures@plos.org).

In the revised draft we used PACE digital diagnostic tool to ensure that figures meet PLOS requirements.

Data Requirements:

Please note that, as a condition of publication, PLOS' data policy requires that you make available all data used to draw the conclusions outlined in your manuscript. Data must be deposited in an appropriate repository, included within the body of the manuscript, or uploaded as supporting information. This includes all numerical values that were used to generate graphs, histograms etc. For an example see here:

[http://www.plosbiology.org/article/info%3Adoi%2F10.1371%2Fjournal.pbio.1001908#s5](http://www.plosbiology.org/article/info%3Adoi%2F10.1371%2Fjournal.pbio.1001908#s5).

We are thankful for providing us these informations. We make available all numerical values data used to generate graphs, PCA, Heat maps or other conclusions outlined in our manuscript. The numerical data used in all figures are included in **Supporting** **Datasets “S3 Numerical datasets used in all figures”** in the revised draft.

Reproducibility:

To enhance the reproducibility of your results, PLOS recommends that you deposit laboratory protocols in protocols.io, where a protocol can be assigned its own identifier (DOI) such that it can be cited independently in the future. For instructions see [http://journals.plos.org/plospathogens/s/submission-guidelines#loc-materials-and-methods](http://journals.plos.org/plospathogens/s/submission-guidelines#loc-materials-and-methods)

N/A

New **References** added to the re-submitted draft

56.	Hangas A, Aasumets K, Kekäläinen NJ, Paloheinä M, Pohjoismäki JL, [Gerhold JM](https://www.ncbi.nlm.nih.gov/pubmed/?term=Gerhold JM%5BAuthor%5D&cauthor=true&cauthor_uid=30169847), [Goffart S](https://www.ncbi.nlm.nih.gov/pubmed/?term=Goffart S%5BAuthor%5D&cauthor=true&cauthor_uid=30169847). Ciprofloxacin impairs mitochondrial DNA replication initiation through inhibition of Topoisomerase 2. Nucleic Acids Res. 2018; 46: 9625-9636. <https://doi.org/10.1093/nar/gky793>.

57.	Raza MF, Yao Z, Bai S, Cai Z, Zhang H. Tephritidae fruit fly gut microbiome diversity, function and potential for applications. B Entomol Res. 2020; 1-15. https://doi.org/10.1017/ S0007485319000853.
